# A Role for PICKLE in the Regulation of Cold and Salt Stress Tolerance in Arabidopsis

**DOI:** 10.3389/fpls.2019.00900

**Published:** 2019-07-09

**Authors:** Rong Yang, Yechun Hong, Zhizhong Ren, Kai Tang, Heng Zhang, Jian-Kang Zhu, Chunzhao Zhao

**Affiliations:** ^1^CAS Center for Excellence in Molecular Plant Sciences, Shanghai Center for Plant Stress Biology, Chinese Academy of Sciences, Shanghai, China; ^2^University of Chinese Academy of Sciences, Beijing, China; ^3^Department of Horticulture and Landscape Architecture, Purdue University, West Lafayette, IN, United States

**Keywords:** PICKLE, cold stress, RNA-seq, chlorophyll, CBF3

## Abstract

Arabidopsis PICKLE (PKL) is a putative CHD3-type chromatin remodeling factor with important roles in regulating plant growth and development as well as RNA-directed DNA methylation (RdDM). The role of PKL protein in plant abiotic stress response is still poorly understood. Here, we report that PKL is important for cold stress response in Arabidopsis. Loss-of-function mutations in the *PKL* gene lead to a chlorotic phenotype in seedlings under cold stress, which is caused by the alterations in the transcript levels of some chlorophyll metabolism-related genes. The *pkl* mutant also exhibits increased electrolyte leakage after freezing treatment. These results suggest that PKL is required for proper chilling and freezing tolerance in plants. Gene expression analysis shows that *CBF3*, encoding a key transcription factor involved in the regulation of cold-responsive genes, exhibits an altered transcript level in the *pkl* mutant under cold stress. Transcriptome data also show that PKL regulates the expression of a number of cold-responsive genes, including *RD29A, COR15A*, and *COR15B*, possibly through its effect on the expression of *CBF3* gene. Mutation in *PKL* gene also results in decreased cotyledon greening rate and reduced primary root elongation under high salinity. Together, our results suggest that *PKL* regulates plant responses to cold and salt stress.

## Introduction

Plants encounter a variety of adverse environmental conditions throughout their life cycles. Cold stress, including chilling (0–15°C) and freezing (<0°C) temperatures (Zhu et al., 2007), is one of the abiotic stresses that greatly limit plant growth and agricultural productivity. Many temperate plants are able to improve their freezing tolerance after being exposed to low, non-freezing temperature (chilling) conditions via a process called cold acclimation (Thomashow, 1999; Chinnusamy et al., 2003). Cold acclimation involves large scale gene expression changes and metabolic alterations (Kreps et al., 2002; Fowler et al., 2005; Hannah et al., 2005). Analyses of cold-responsive genes led to the discovery of three C-REPEAT BINDING FACTOR (CBF) genes, named *CBF1, CBF2*, and *CBF3*. The transcript levels of these three *CBF* genes are rapidly induced under cold stress (Stockinger et al., 1997; Gilmour et al., 1998). Constitutive overexpression of *CBF1, CBF2*, or *CBF3*gene increases the expression of many cold-responsive (COR) genes, including *COR15, COR47, COR78*, and *KIN1*, and subsequently increases freezing tolerance in Arabidopsis (Jaglo-Ottosen et al., 1998; Liu et al., 1998; Gilmour et al., 2004). Simultaneously mutating these three *CBF* genes attenuates the transcript levels of COR genes and results in hypersensitivity to freezing (Zhao et al., 2016). The expression of the three *CBF* genes under cold stress is regulated by several upstream transcription factors, including EIN3, MYB15, CAMTAs, and ICE1 (Chinnusamy et al., 2003; Doherty et al., 2009; Zhao et al., 2015). Drought and salinity are another two abiotic stress factors that adversely affect the growth and development in plants. It has been reported that many genes, such as *RD29A* and *RD29B*, are commonly induced by drought, high-salinity, and cold stress, while some genes are induced under a specific abiotic stress condition (Rabbani et al., 2003).

PICKLE (PKL) is a component of the Mi-2/CHD3 subfamily of ATP-dependent chromatin remodelers (Flaus et al., 2006; Ho et al., 2013). Chromatin remodeling factors are required for changing the conformation of nucleosomes and chromatin by utilizing energy generated from ATP hydrolysis. PKL was identified that regulates many plant development processes, including embryonic development, seed germination, root meristem activity, and hypocotyl cell elongation during skotomorphogenesis (Ogas et al., 1999; Fukaki et al., 2006; Perruc et al., 2007; Aichinger et al., 2011; Jing et al., 2013). In many developmental processes, the CHD3-type chromatin remodeling factors are involved in transcriptional co-activation, but the PKL protein functions as a transcriptional repressor in Arabidopsis (Saether et al., 2007; Lai and Wade, 2011). The PKL protein has a nucleosome remodeling activity that is involved in the trimethylation of histone H3 lysine 27 (H3K27me3), the repressive histone mark connected with the regulation of tissue-specific genes (Zhang et al., 2008, 2012). In our previous study, we found that PKL is involved in the RNA-directed DNA methylation (RdDM) pathway (Yang et al., 2017). Mutations in the *PKL* gene led to severe developmental phenotypes, including small and curled leaves and siliques (Yang et al., 2017). Based on the results from the analysis of DNA methylomes, small RNAs, and transcriptomes in *pkl-1* mutants, we proposed that PKL produces a chromatin environment at RdDM target regions via its nucleosome remodeling activity, and subsequently affects non-coding RNA transcription, DNA methylation, and transcriptional silencing.

A few studies have shown that disruption of the RdDM pathway results in defects in abiotic stress responses (He et al., 2009b; Kanno et al., 2010). For example, mutation in the *RDM4* gene, which is involved in the RdDM pathway, causes increased sensitivity to cold stress in Arabidopsis. RDM4 regulates the expression of COR genes via a CBF-dependent pathway (Chan et al., 2016). It was reported that the *pkl* mutant is hypersensitive to abscisic acid (ABA) in terms of seed germination, which is caused by the constitutively increased transcript levels of *ABI3* and *ABI5* genes (Perruc et al., 2007; Belin and Lopez-Molina, 2008). Recently, it was shown that the *hrb2*, another allele of *pkl* mutant in Ws background, exhibits reduced water loss and increased sensitivity to ABA (Kang et al., 2018). Besides, *pkl* mutation results in reduced hypocotyl elongation under warm temperature (28°C) (Zha et al., 2017). So far, the roles of PKL in the response to other abiotic stresses, such as cold and salinity are still poorly understood, and the mechanisms underlying the regulatory role of PKL in abiotic stress tolerance are still largely unknown.

In this study, we present evidences for a role of PKL in cold and salt stress tolerance. Disruption of the *PKL* gene led to hypersensitivity to both chilling and freezing, which is associated with the altered transcript abundance of some cold-responsive genes. Transcriptomic profiling of cold-treated wild-type plants and *pkl* mutants revealed that PKL regulates the expression of some COR genes by modulating the expression of *CBF3* gene. Together, our study establishes a genetic role for PKL in cold-responsive gene regulation and cold tolerance. Besides, our data also support a positive role of PKL in the regulation of salt stress response.

## Materials and Methods

### Plant Materials and Growth Conditions

The *pkl-1* (Col-0 ecotype), *rdm18-1*, and *rdm18-2* (C24 ecotype) mutant alleles used in this study were the same as those reported previously (Yang et al., 2017). Plants were grown in growth chambers at 22°C with 16 h-light/8 h-dark cycle. For the chilling treatment, plants were grown in growth chamber at 4 °C with a 16 h-light/8 h-dark cycle, and the control plants were grown in chamber at 22°C with the same light condition.

### Measurement of Chlorophyll Content

For the chilling treatment, the 5-day-old seedlings were transferred to incubator with 4°C and incubated for 30 days. The leaf tissues of the control (22°C for 16 days) and chilling plants were ground into powder using liquid nitrogen, and then chlorophyll pigments were extracted using 80% ice-cold acetone according to the method described previously (Ni et al., 2009). The chlorophyll content was quantified by using Varioskan Flash spectrophotometer (Thermo Scientific).

### Quantitative Real-Time RT-PCR

Quantitative real-time RT-PCR (qRT-PCR) was performed following the protocol described previously (He et al., 2009a). For the gene expression analysis, the leaves of 10-day-old plants were subjected to cold treatment (0, 3, and 24 h at 4°C, respectively). Total RNAs were extracted using RNeasy Mini Kit (Qiagen, Germany). Genomic DNAs were removed using Turbo DNase (Ambion). The RNA without genomic DNA was used for the cDNA synthesis with a Superscript III First Strand Synthesis Kit (Invitrogen, United States) according to manufacturer’s instructions. The derived cDNA was diluted to 5–10 ng/μL and 5 μL was used as a template for qRT-PCR analysis. The primers used for qRT-PCR analysis are listed in Supplementary Table S5.

### Electrolyte Leakage Assay

Eletrolyte leakage assay was performed to test the freezing tolerance. The electrolyte leakage assay was performed following the method used previously (Zhao et al., 2016). Three-week-old plants with or without cold acclimation (4°C, 7 days) were applied with a freezing treatment. A developed rosette leaf was detached from the plants and placed in a tube with 100 μL deionized water. At the same time, a small size of ice chip was placed to each tube, which was incubated in a freezing bath (model 1187; VWR Scientific) immediately. For every sample, three biological replicates were performed. After all the tubes were placed in the bath, the freezing process started with 1°C decrements every 30 min, and the tubes were moved from the freezing bath when the temperature reaches to -2, -4, -5, -6, -7, -8, -9, -10, and -11°C. The removed tubes were placed on ice for a while and then shaken overnight at room temperature. After shaking, the conductivity of solution was measured. Then, the solution was autoclaved and shaken for 3 h, and the conductivity of solution was measured again. Finally, the ratio of conductivity before and after autoclaving was calculated.

### Transcriptome Sequencing

For the transcriptome sequencing, the total RNAs were extracted from 10-day-old wild type and *pkl-1* seedlings treated with 4°C for 0, 3, and 24 h using TRIzol reagent (Life Technologies, United States). The total RNAs were sent to Core Facility for Genomics of Shanghai Center for Plant Stress Biology (PSC) for transcriptome sequencing by Illumina HiSeq2500. The mRNAs were enriched by poly T. RNA library was prepared and the paired end sequencing was performed using specific-sequencing reagents (Illumina, United States) based on the manufacturer’s instructions.

### Analysis of RNA-Seq Data

Data analysis was performed as previously described (Chen et al., 2016; Yang et al., 2017). After the adapter sequences and low-quality bases (*q* < 30) were trimmed, the clean reads were obtained and mapped to the TAIR10 reference genome using TopHat. Mapped read counts of each gene were generated by the cuffnorm command and the differentially expressed genes were analyzed using cuffdiff in Cufflinks. For the differentially expressed genes, a threshold of *q*-value < 0.05, and at least 2-fold change was used in the study. The GO enrichment analysis was performed by AgriGO (Du et al., 2010).

### Germination Assay

For the germination assay, we calculated the cotyledon greening rate of the plants. About 150 seeds were surface-sterilized and sown on half-strength MS medium or medium supplemented with NaCl (150 mM), ABA (0.5 μM), or mannitol (200 mM). All of the seeds were vernalized for 3 days and then grown in an incubator at 22°C. The cotyledon greening rate was calculated after 6 days. For measurement of root length of seedlings, the seeds were sown on half-strength MS medium and grown for 3 days before the seedlings were transferred to half-strength MS medium or medium supplemented with NaCl, ABA, or mannitol. Ten-day-old seedlings were used for the measurement of root length.

## Results

### Mutation of the *PKL* Gene Leads to Hypersensitivity to Chilling Stress

**FIGURE 1:**
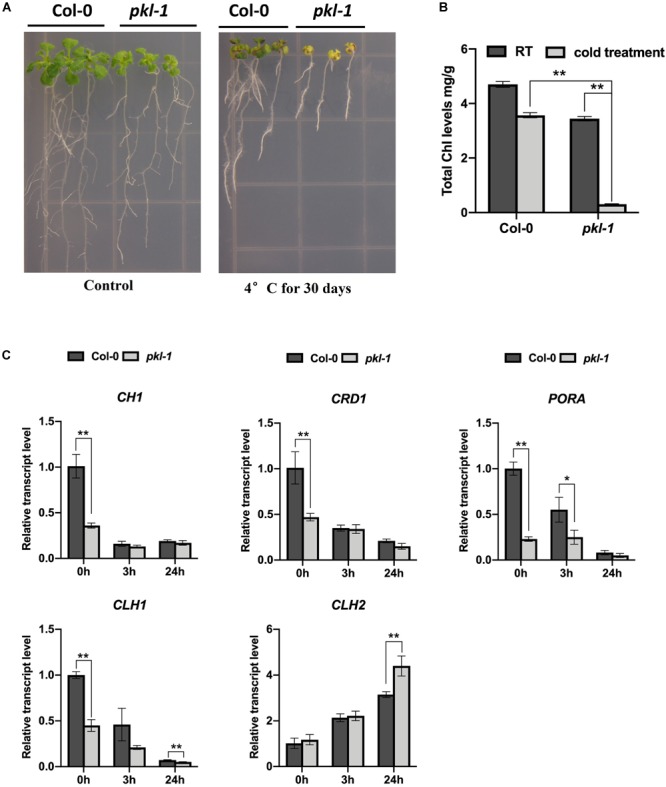
Arabidopsis *pkl-1* mutant is hypersensitive to chilling. **(A)** Phenotype of the *pkl-1* mutants at room temperature (22°C, left panel) and after chilling treatment (4°C, right panel). For the chilling treatment, 5-day-old seedlings grown on a half-strength MS medium were transferred to an incubator with 4°C for 30 days. The experiment was repeated more than three times with similar results. **(B)** Quantification of chlorophyll content in the 10-day-old seedlings of the wild type and *pkl-1* seedlings under room temperature (22°C) or after chilling treatment (4°C) for 30 days. Chl, chlorophyll. **(C)** qRT-PCR analysis of the genes that are associated with chlorophyll biosynthesis and degradation before and after cold treatment (4°C). *ACTIN2* served as the internal control. Error bars in **(B,C)** indicate the SD of three biological replicates. Asterisks represent significant differences between the wild type and *pkl-1* mutant (^∗^*p* < 0.05 and ^∗∗^*p* < 0.01, two-tailed *t*-tests).

It has been shown that *pkl* mutants exhibit multiple developmental defects, including small and curled leaves, and short and curled siliques (Yang et al., 2017). Here, we found that when seedlings were exposed to a chilling condition (4°C), the leaves of the *pkl-1* mutant were much yellower than those of the Col-0 wild type (Figure 1A). There was no obvious difference between the wild type and *pkl-1* seedlings in terms of the color of leaves under normal growth temperature (22°C) (Figure 1A). Another two alleles of *pkl* mutant, *rdm18-1* and *rdm18-2*, which are both in the C24 ecotype (Yang et al., 2017), also exhibited a chlorotic phenotype when grown under chilling conditions (Supplementary Figure S1). These results suggest that PKL/RDM18 is involved in the response to chilling stress.

To investigate whether the chlorotic phenotype of the *pkl/rdm18* mutants after being exposed to chilling conditions is due to defects in chlorophyll accumulation, we measured the content of chlorophylls in the wild type and *pkl-1* mutants after cold treatment for 30 days. The result showed that the chlorophyll content in the *pkl-1* mutant was slightly reduced under normal conditions but was substantially decreased under chilling conditions compared to the wild type plants (Figure 1B), which suggests that PKL is required for the accumulation of chlorophylls under chilling conditions.

The chlorophyll metabolic pathway includes three different phases: synthesis of chlorophyll, chlorophyll cycle that involves an interconversion between chlorophylls *a* and *b*, and degradation of chlorophyll *a* (Tanaka and Tanaka, 2006; Masuda and Fujita, 2008; Hortensteiner, 2013). The *CH1, CRD1, PORA, CLH1*, and *CLH2* genes are required for chlorophyll metabolism (Hortensteiner, 2013; Ohmiya et al., 2014). We examined the transcript levels of these genes in the wild type and *pkl-1* mutants before and after cold treatment using quantitative real-time RT-PCR (qRT-PCR). The expressions of *CH1* and *CRD1* genes in the *pkl-1* mutants were reduced under normal conditions but were not affected after cold treatment compared with that in the wild type plants. The transcript levels of *PORA* and *CLH1* genes were decreased under both normal and cold stress conditions (Figure 1C). The expression level of the *CLH2* gene, which functions in chlorophyll degradation, was increased in the *pkl-1* mutant after cold treatment for 24 h compared with the wild type plants (Figure 1C). These results suggest that alterations in the transcript levels of chlorophyll metabolism-associated genes, particularly the cold-upregulation of the chlorophyll degradation gene *CLH2*, are likely the causes of the chlorotic phenotype of the *pkl-1* mutant under cold stress.

### The *pkl* Mutants Are Defective in Cold Acclimation

**FIGURE 2:**
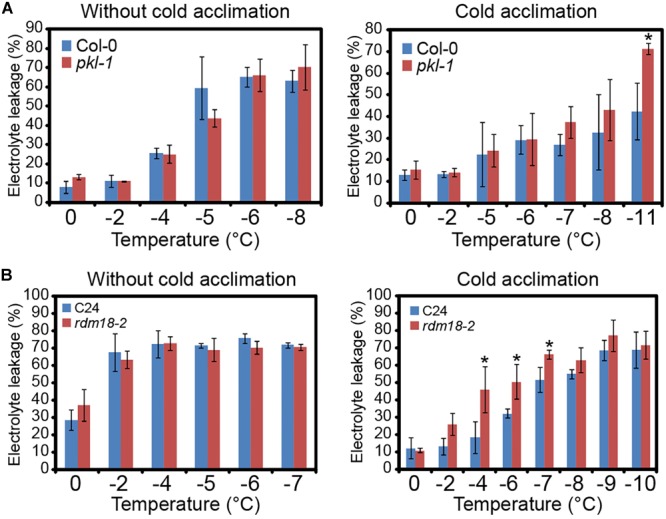
*pkl*/*rdm18* mutants are defective in cold acclimation. **(A)** Three-week-old wild type (Col-0 ecotype) and *pkl-1* mutant without (Left) or with (Right) cold acclimation (4°C) were subjected to freezing treatment. The electrolyte leakage was assessed by measuring conductivity. **(B)** Electrolyte leakage assays were performed for the 3-week-old wild type (C24 ecotype) and *rdm18-2* without (Left) or with (Right) cold acclimation (4°C). Error bars represent the SD of three biological replicates. Asterisks represent significant differences between the wild type and mutants (^∗^*p* < 0.05, two-tailed *t*-tests).

To examine the role of PKL protein in freezing stress tolerance, we performed electrolyte leakage (EL) assay. When plants were subjected to freezing without cold acclimation, the EL values were similar between the wild type plants and *pkl-1* mutants (Figure 2A). However, when seedlings were subjected to cold acclimation (4°C treatment) before being exposed to freezing treatment, the EL value of the *pkl-1* mutant was higher than those of the wild type (Figure 2A), suggesting that *pkl-1* is hypersensitive to freezing. The *rdm18-2* allele in C24 ecotype also exhibited higher EL values than the wild type under freezing at -4, -6, and -7°C after cold acclimation (Figure 2B). These results indicate that PKL is required for proper freezing tolerance that depends on cold acclimation.

### PKL Regulates the Expression of Stress-Responsive Genes

**FIGURE 3:**
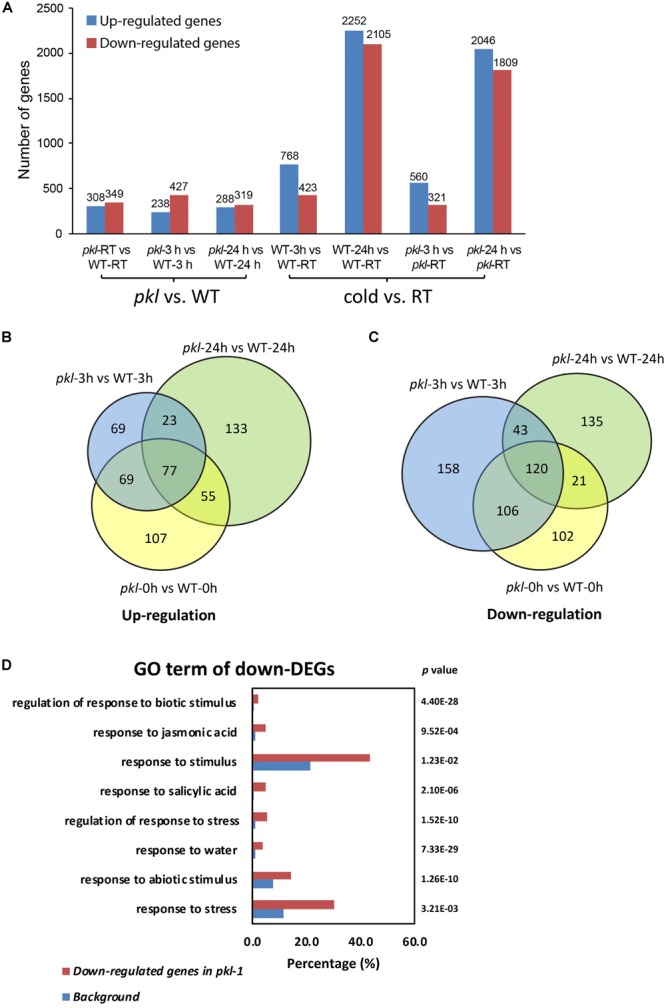
RNA-seq analysis of the differentially expressed genes in the *pkl-1* mutant before and after cold treatment. 10-day-old seedlings of the wild type and *pkl-1* treated with or without cold (4°C) were subjected to RNA-Seq analysis. Three biological replicates were performed for each sample at each time point. **(A)** The number of genes that were differentially expressed in the *pkl-1* mutant compared with the wild type, and the number of genes that were up-regulated or down-regulated in the wild type and *pkl-1* after cold treatment. **(B,C)** Comparison of genes that were up-regulated **(B)** and down-regulated **(C)** in the *pkl* mutant at each time point after cold treatment. **(D)** GO term enrichment analysis of genes that were down-regulated in the *pkl-1* mutant after cold treatment.

To determine whether some cold-responsive genes may be regulated by PKL, the wild type and *pkl-1* seedlings after 4°C treatment for 0, 3, and 24 h were collected for RNA sequencing. Three independent biological replicates were performed for each sample at each time point. We first analyzed the genes that were differentially expressed after cold treatment in wild type plants. 768 genes were up-regulated at 3 h and 2,252 genes were up-regulated at 24 h, while 423 genes were down-regulated at 3 h, and 2,105 genes were down-regulated at 24 h in the wild type (Figure 3A, Supplementary Figure S2 and Supplementary Table S1). RNA-Seq data revealed that 657, 665, and 607 genes were differentially expressed in the *pkl-1* mutant compared with the wild type at 0, 3, and 24 h after cold treatment (4°C), respectively (Figure 3A). Among these genes, 238 genes were up-regulated and 427 genes were down-regulated after cold treatment for 3 h, and 288 genes were up-regulated and 319 genes were down-regulated after cold treatment for 24 h in the *pkl-1* mutant (Figure 3A). 100 genes were up-regulated and 163 genes were down-regulated in the *pkl-1* mutant at both 3 h and 24 h after cold treatment (Figure 3B,C).

Gene ontology (GO) term enrichment analysis for down-regulated genes in the *pkl-1* mutants after cold treatment were conducted. The results showed that the down-regulated genes in the *pkl-1* mutant were enriched in “response to water” (*P*-value = 7.33E-29), “response to abiotic stimulus” (*P*-value = 1.26E-10), “response to stress” (*P-*value = 3.21E-03), and “response to stimulus” (*P*-value = 1.23E-02) (Figure 3D), suggesting that PKL is involved in the regulation of stress-responsive genes.

**FIGURE 4:**
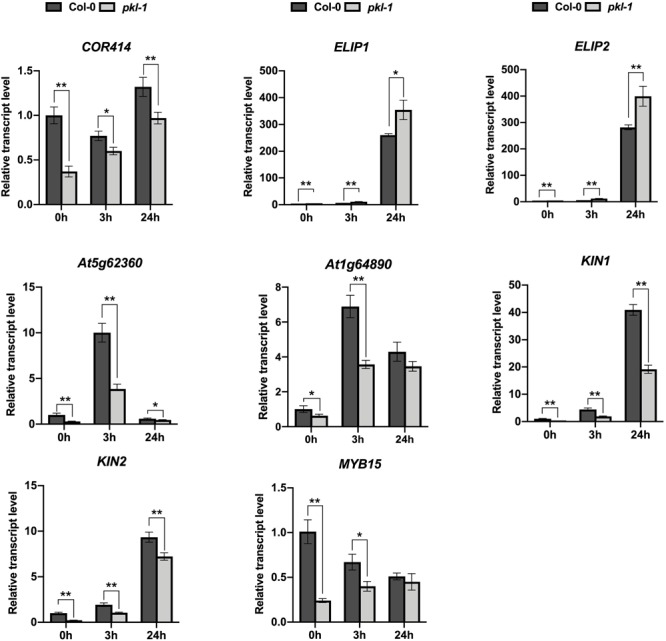
Analysis of the expression of PKL-regulated genes. qRT-PCR analysis of the genes after cold treatment (4°C) for 0, 3, and 24 h in the 10-day-old seedlings of the wild type and *pkl-1* mutant. *ACTIN2* was served as the internal control. Error bars represent the SD of three biological replicates. Asterisks represent significant differences between the wild type and *pkl-1* mutant (^∗^*p* < 0.05 and ^∗∗^*p* < 0.01, two-tailed *t*-tests).

We analyzed the expression of the chlorophyll metabolism-related genes, including *CH1, CRD1, PORA, CLH1*, and *CLH2*, in our RNA-Seq data (Supplementary Table S2), and found that the expression patterns of these genes obtained from the RNA-Seq data were consistent with the qRT-PCR results as shown in Figure 1C. These results indicated that our RNA-Seq data were reliable. Further, we performed qRT-PCR to test the expression of eight cold-responsive genes that were differentially expressed in the *pkl-1* mutant compared with the wild type. These genes include two up-regulated genes (*ELIP1* and *ELIP2*) and six down-regulated genes (*COR414*, invertase (At5g62360), transporter (At1g64890), *KIN1, KIN2*, and *MYB15*) (Supplementary Table S3). Consistent with the RNA-seq data, qRT-PCR results showed that the *ELIP1* and *ELIP2* genes were up-regulated, while the *COR414*, invertase (At5g62360), *KIN*, transporter (At1g64890), *KIN2*, and *MYB15* genes were down-regulated in the *pkl-1* mutant compared to the wild type (Figure 4), suggesting that PKL is involved in the regulation of cold-responsive genes.

### PKL May Regulate Cold-Responsive Genes by Affecting the Expression of *CBF3* Gene

**FIGURE 5:**
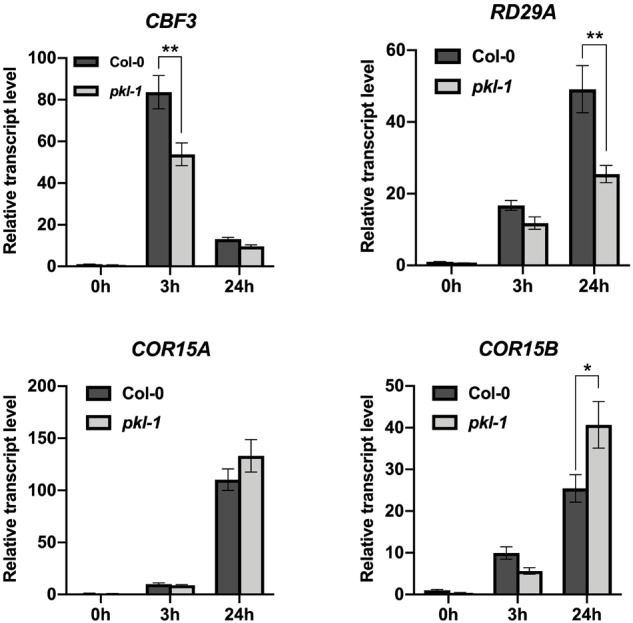
Analysis of the expression of *CBF3* and COR genes in the *pkl* mutant. Ten-day-old seedlings of the wild type and *pkl-1* were placed at 4°C for 0, 3, and 24 h. The transcript levels of *CBF3, RD29A, COR15A*, and *COR15B* were assessed by qRT-PCR. *ACTIN2* served as the internal control. Error bars represent the SD of three biological replicates. Asterisks represent significant differences between the wild type and *pkl-1* mutant (^∗^*p* < 0.05 and ^∗∗^*p* < 0.01, two-tailed *t*-tests).

We further investigated whether PKL may regulate the cold-responsive genes via the CBF pathway. Our RNA-seq data showed that the transcript levels of *CBF1* and *CBF2* genes were not affected, but the transcript level of *CBF3* gene was down-regulated in the *pkl-1* mutant after cold treatment for 3 h (Supplementary Table S3). Compared with the wild type plants, many CBF-regulated genes were down-regulated in the *pkl-1* mutant (Supplementary Table S3). qRT-PCR analysis confirmed that the cold-induced expression of *CBF1* and *CBF2* genes was not affected in the *pkl-1* mutants, whereas the transcript level of *CBF3* gene was reduced in the *pkl-1* mutants compared with the wild type plants after cold treatment for 3 h (Figure 5). For the COR genes, *RD29A* was down-regulated at 3 and 24 h after cold treatment, while the *COR15A* and *COR15B* genes were down-regulated at 3 h but up-regulated at 24 h after cold treatment in the *pkl-1* mutant compared with the wild type plants (Figure 5). The expression patterns for *CBF* and COR genes in the *rdm18* mutants after cold treatment were similar to those observed in the *pkl-1* mutant (Supplementary Figure S3). These results suggest that the PKL/RDM18 is involved in the regulation of COR genes, possibly through its effect on the expression of *CBF3* gene. To further verify the association between the PKL and the CBF pathway, we compared genes that are differentially expressed in the *pkl-1* mutant and in the *CBF3* overexpressing plants reported by Chan et al. (2016). We found that 50 genes that are up-regulated in the *CBF3* overexpressing plants, were down-regulated in the *pk1-1* mutant (Supplementary Table S4).

### Mutation in the *PKL* Gene Leads to Hypersensitivity to Salt Stress

**FIGURE 6:**
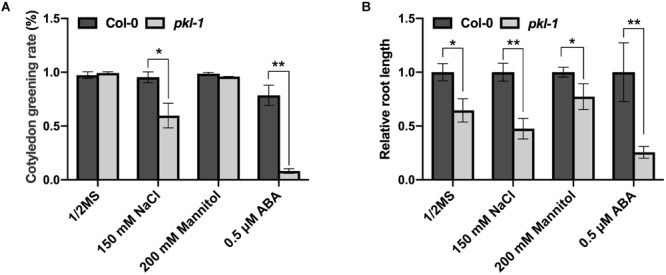
Mutation of *PKL* gene leads to hypersensitivity to high salinity. **(A)** Cotyledon greening rate of the wild-type and *pkl-1* plants grown on half-strength MS medium or half-strength MS medium supplemented with 150 mM NaCl, 200 mM mannitol, or 0.5 μM ABA. The cotyledon greening rate was assessed after growth for 6 days. Error bars indicate the SD of three biological replicates. **(B)** Five-day-old seedlings of wild type and *pkl-1* were transferred to half-strength MS medium or half-strength MS medium supplemented with NaCl (150 mM), mannitol (200 mM), or ABA (0.5 μM). The root length of the wild type plants on each medium was set to 1. Error bars indicate the SD of four biological replicates. Asterisks represent significant differences between the wild type and *pkl-1* mutant (^∗^*p* < 0.05 and ^∗∗^*p* < 0.01, two-tailed *t*-tests).

To examine whether the *PKL* gene is involved in the response to other abiotic stresses, we examined the phenotypes of the *pkl-1* mutant grown on Murashige and Skoog (MS) agar medium or MS medium supplemented with ABA, salt, or mannitol. On MS medium, no difference was observed for cotyledon greening rate between the wild type and *pkl-1* mutant. On the MS medium supplemented with ABA (0.5 μM), the cotyledon greening rate of *pkl-1* mutants was substantially lower than that of the wild type (Figure 6A), which is consistent with previous reports (Perruc et al., 2007; Belin and Lopez-Molina, 2008). On the MS media supplemented with NaCl (150 mM), but not with mannitol, the cotyledon greening rate was also decreased in the *pkl-1* mutant compared with the wild type (Figure 6A). Consistently, primary root elongation of the *pkl-1* mutant was more inhibited by applying NaCl or ABA but was only slightly more affected by applying mannitol to the media compared with that of the wild type (Figure 6B). These results suggest that PKL is involved in the regulation of salt stress tolerance and ABA response.

## Discussion

Previous studies have shown that the chromatin remodeling factor PKL is involved in the regulation of plant development and RdDM in Arabidopsis (Yang et al., 2017), but the role of PKL in abiotic stress response is still largely unknown. Here, we found that PKL is involved in the regulation of plant responses to cold and salt stresses. Mutation in *PKL* gene results in a chlorotic phenotype under chilling conditions and an increased electrolyte leakage under freezing stress, suggesting that PKL protein is required for both chilling and freezing tolerance. Gene expression analysis revealed that the increased sensitivity of the *pkl-1* mutant to cold is likely due to reduced expression of *CBF3* gene.

It has been shown that a large number of cold-responsive proteins are located in the chloroplasts in rice (Leister, 2003; Taylor et al., 2009; Li et al., 2018). Knock-out of some chloroplast protein-encoding genes leads to chilling-hypersensitive phenotype (Wang et al., 2016), while overexpression of several stress-inducible chloroplast protein-encoding genes improves cold tolerance (Zhong et al., 2013; Kong et al., 2014; Li et al., 2018). These results suggest that biological processes in the chloroplast are critical for chilling tolerance. Our study showed that the *pkl-1* mutant displayed a chlorotic phenotype under cold stress, and the expression of some chlorophyll metabolism-related genes was affected in the *pkl-1* mutant, suggesting that PKL is required for the regulation of chlorophyll metabolism under cold stress. However, whether PKL directly regulates the genes that are associated with the metabolism of chlorophyll needs further investigation.

RNA-Seq analysis revealed that a large number of genes were down-regulated in the *pkl-1* mutant after cold treatment, suggesting the important role of PKL in cold stress response. These down-regulated genes were enriched in the categories “response to jasmonic acid” and “response to salicylic acid,” indicating that PKL regulates JA and SA pathways under cold stress. It has been shown that activation of JA pathway can enhance cold tolerance in plants (Chen et al., 2019). Therefore, we speculate that the defect in the JA pathway could be one of the causes of the cold-hypersensitivity in the *pkl-1* mutant. RNA-Seq data also showed that many abiotic stress-responsive genes were down-regulated in the *pkl-1* mutant, which confirms the role of PKL protein in the regulation of abiotic stress tolerance.

Overexpression of *CBF* genes in transgenic plants leads to increased expression of many cold-responsive (COR) genes (Park et al., 2015), whereas mutation of all the three *CBF* genes substantially reduces the expression of CBF regulon genes and results in hypersensitivity to freezing stress (Zhao et al., 2016). These results suggested that CBF-mediated pathways are important for freezing tolerance. Our results showed that the cold-induced expression of *CBF1* and *CBF2* genes was not decreased, but the *CBF3* gene was significantly down-regulated in the *pkl-1* mutant at 3 h after cold stress treatment, suggesting that PKL may regulate the expression of *CBF3* gene and thus modulate the expression of some downstream cold-responsive genes to increase cold tolerance. It has been shown that *CBF* genes are involved in the regulation of several cold-responsive genes that are associated with chloroplast process (Zhao et al., 2016), which suggests that the chlorotic phenotype of the *pkl-1* mutant is likely due to reduced *CBF3* gene expression under cold stress.

The molecular mechanism underlying the role of PKL in the regulation of cold and salt stresses remains to be elucidated. *PKL* protein is an ATP-dependent chromatin remodeler that is involved in the regulation of nucleosome position. One hypothesis to explain the role of PKL in cold stress tolerance is that the cold-induced expression of *CBF3* gene or other cold-responsive genes, which are down-regulated in the *pkl-1* mutant, depends on histone state on their promoters. The histone modification changes in the promoter region of these cold-responsive genes may affect their expression under cold stress and thus affect cold stress tolerance. Another hypothesis is that changes in nucleosome conformation or composition in the *pkl-1* mutant may affect the access of transcription factors to the promoters of cold-responsive genes. Whether the histone level or nucleosome organization is affected in the promoter of cold-responsive genes remains to be explored.

Under high salinity, cotyledon greening rate and primary root elongation were both decreased in the *pkl-1* mutant compared with the wild type, indicating that PKL is required for the tolerance to salt stress. Our study also showed that mutation in *PKL* gene results in the inhibition of seed germination under high concentrations of ABA, which is consistent with previous reports (Perruc et al., 2007; Belin and Lopez-Molina, 2008). It is well-known that PKL is a significant regulator in plant growth and development (Ogas et al., 1997). The pleiotropic functions of PKL suggest that PKL-mediated regulatory modules are important for coordinating developmental programs and environmental stress responses to optimize plant growth under stress.

In summary, our study showed that the CHD3 protein PKL plays a role in the regulation of cold stress response likely via the regulation of chlorophyll accumulation under cold stress. Our results suggest that PKL may regulate cold stress response, at least partially, via a CBF3-mediated pathway. Besides, our results reveal that the PKL gene is also involved in the regulation of salt stress tolerance. These findings advance our understanding of the biological functions of PKL in plant stress responses.

## Data Availability

The datasets generated for this study are available in the Gene Expression Omnibus (GEO) (https://www.ncbi.nlm.nih.gov/geo/) under the accession code GSE127819.

## Author Contributions

RY and J-KZ designed the project. RY and CZ performed the experiments and discussed the results. RY, CZ, YH, ZR, KT, and HZ analyzed the data. RY, CZ, and J-KZ wrote the manuscript.

## Conflict of Interest Statement

The authors declare that the research was conducted in the absence of any commercial or financial relationships that could be construed as a potential conflict of interest.
