# Supplementary material for: A Role for PICKLE in the Regulation of Cold and Salt Stress Tolerance in Arabidopsis
Source: Front Plant Sci. 2019 Jul 9;10:900. doi: 10.3389/fpls.2019.00900 (PMC6633207; doi:10.3389/fpls.2019.00900)
Supplement: Supplementary file 1 [file Data_Sheet_1.docx]

## Supplementary Figures


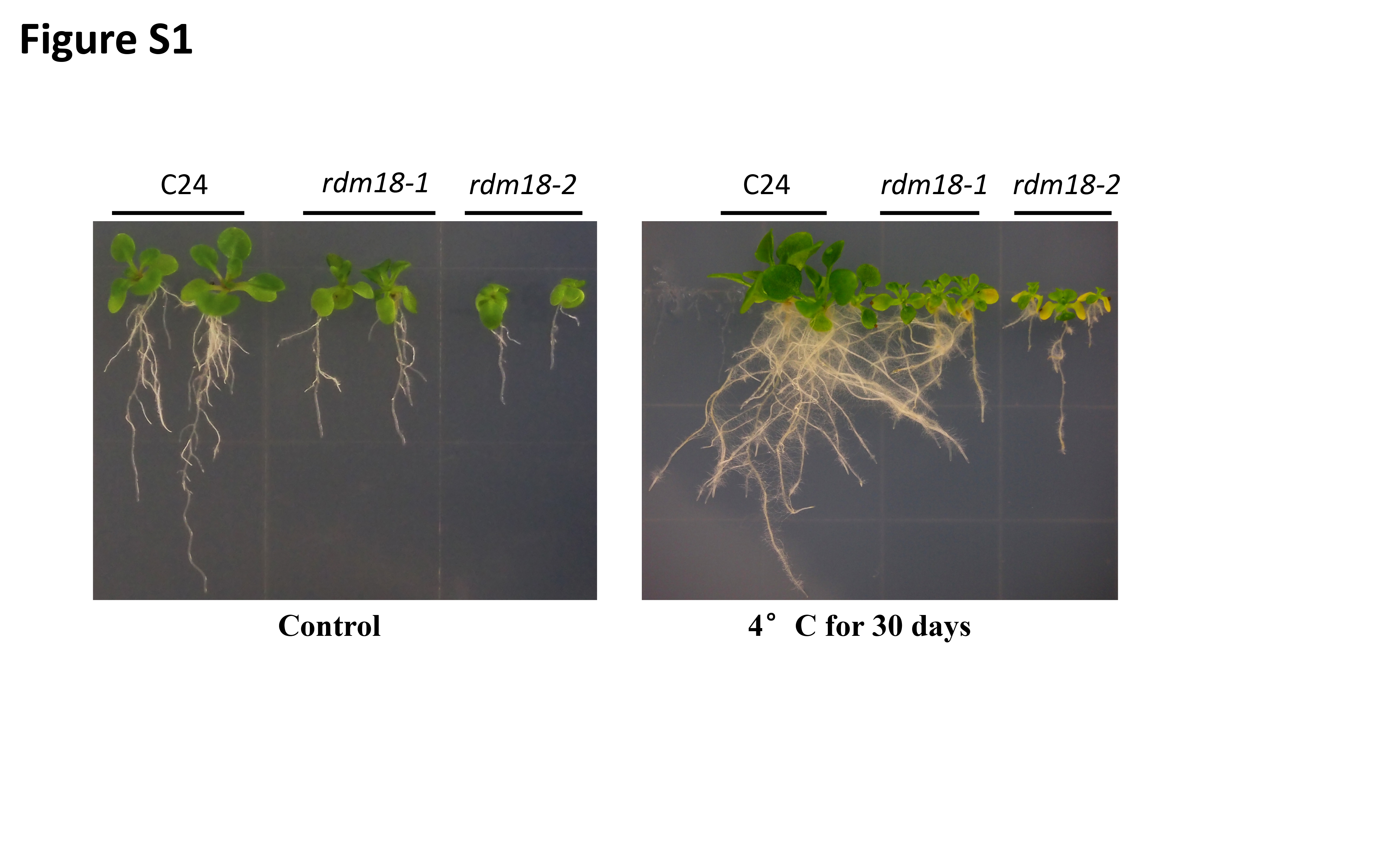


**Supplementary Figure 1**. ***rdm18-1* and *rdm18-2* mutants are sensitive to chilling.**

Phenotype of the *rdm18-1* and *rdm18-2* mutants at room temperature (left) and after chilling treatment (right). For chilling treatment, five-day-old seedlings grown on a MS medium were transferred to an incubator with 4°C for 30 days.


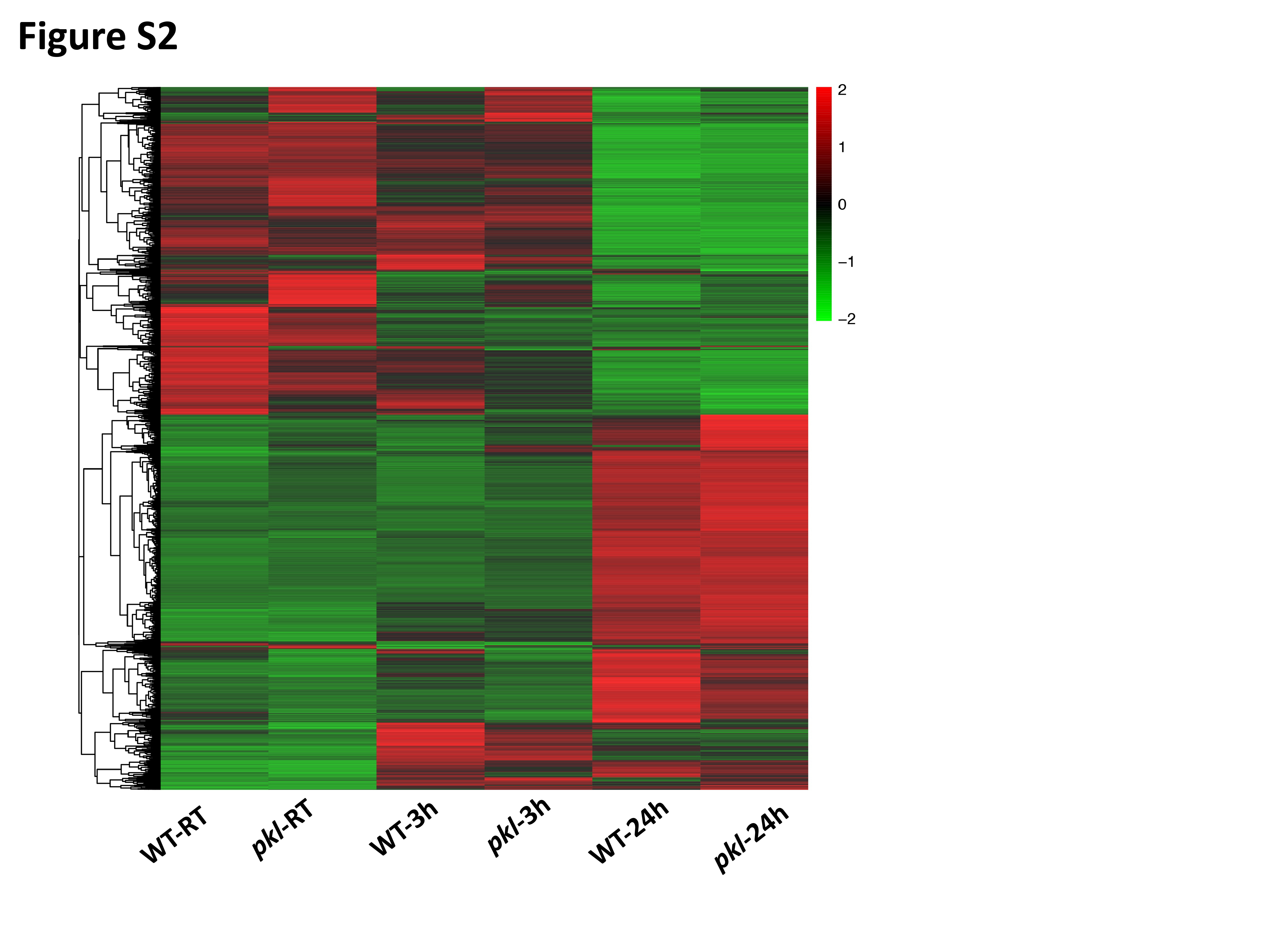


**Supplementary Figure 2**. Heat-map shows the genes that were up-regulated or down-regulated in the *pkl* mutant after being exposed to 4°C for 3 h and 24 h.


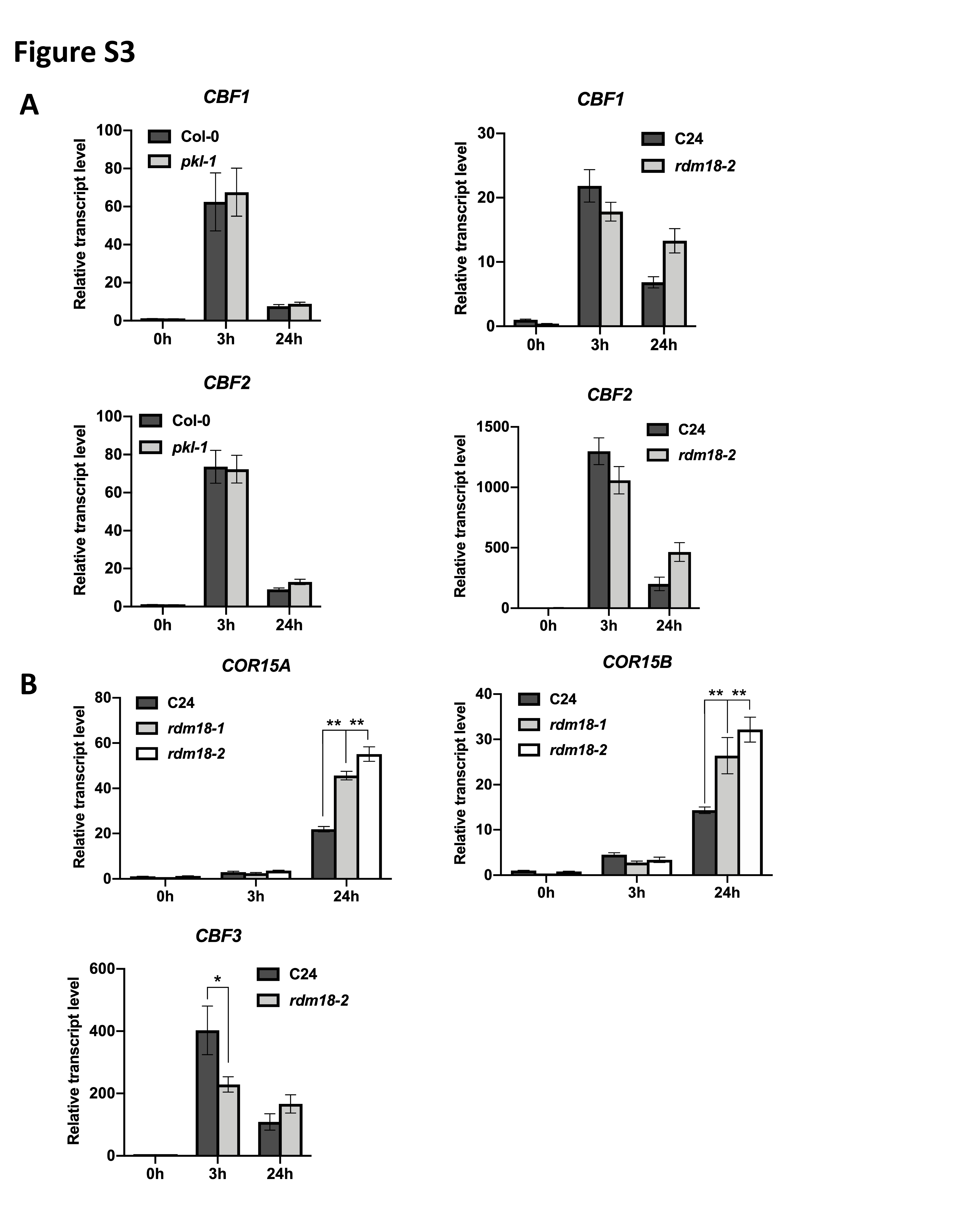


**Supplementary Figure 3**. **Analysis of the expression of *CBFs* and CBF-regulated genes.** (A) Transcript levels of *CBF1* and *CBF2* genes in the *pkl-1* mutant (Col-0 background) and *rdm18-2* (C24 background) mutant were detected by qRT-PCR. *ACTIN2* was used as the internal control. (B) qRT-PCR analysis of the *COR15A*, *COR15B*, and *CBF3* genes in the wild type (C24 ecotype), *rdm18-1*, and *rdm18-2* after cold treatment (4°C) for 0, 3, and 24 h. *ACTIN2* was served as the internal control. Error bars represent the SD of three biological replicates. Asterisks represent significant differences between the wild type and mutants (*p <0.05 and **p <0.01, two-tailed *t*-tests).
